# *In vitro* and *in silico* β-lactamase inhibitory properties and phytochemical profile of *Ocimum basilicum* cultivated in central delta of Egypt

**DOI:** 10.1080/13880209.2022.2127791

**Published:** 2022-10-13

**Authors:** Nagwa A. Shoeib, Lamiaa A. Al-Madboly, Amany E. Ragab

**Affiliations:** a Department of Pharmacognosy, Tanta University, Tanta, Egypt; b Department of Pharmaceutical Microbiology, Tanta University, Tanta, Egypt

**Keywords:** Methyl cinnamate, antimicrobial

## Abstract

**Context:**

Some studies reported the chemical content and antimicrobial properties of *Ocimum basilicum* L. (Lamiaceae), relevant to the ecological variations in some areas of Egypt and other countries, yet no research was conducted on the plant cultivated in the central delta region of Egypt. Also, no previous data reported on inhibition of β-lactamases by *O. basilicum.*

**Objective:**

To assess β-lactamases inhibition by *O. basilicum* extracts and the individual constituents.

**Materials and methods:**

Dried aerial parts of *O. basilicum* were extracted by hydrodistillation for preparation of essential oil and by methanol for non-volatile constituents. Essential oil content and the methanol extract were analysed by GC–MS and UPLC-PDA-MS/MS, respectively. Methyl cinnamate was isolated and analysed by NMR. Broth microdilution method was used to investigate the antimicrobial against resistant clinical isolates of *Escherichia coli* identified by double disc synergy, combination disc tests and PCR. The most active oil content was further tested with a nitrocefin kit for β-lactamase inhibition and investigated by docking.

**Results:**

*O. basilicum* was found to contain methyl cinnamate as the major content of the essential oil. More interestingly, methyl cinnamate inhibited ESBL β-lactamases of the type CTX-M. The *in vitro* IC_50_ using nitrocefin kit was 11.6 µg/mL vs. 8.1 µg/mL for clavulanic acid as a standard β-lactamase inhibitor.

**Discussion and conclusions:**

This is the first study to report the inhibitory activity of *O. basilicum* oil and methyl cinnamate against β-lactamase-producing bacteria. The results indicate that methyl cinnamate could be a potential alternative for β-lactamase inhibition.

## Introduction

The misuse of antibiotics particularly β-lactams resulted in the emergence of multi-drug resistant organisms; therefore, controlling infectious diseases encounters many problems. Resistance of *Escherichia coli* to β-lactams antibiotics is usually triggered by intrinsic β-lactamases that are able to hydrolyse the β-lactam ring of such molecules (Fam et al. 2011). To solve this problem, extended spectrum cephalosporins were initially introduced as therapeutic antibiotics in the mid-1980s; however, extended spectrum β-lactamases (ESBLs) have emerged in several hospitals all over the world (Fam et al. 2011). Such enzymes confer resistance to cephalosporins as well as related oxyimino-β-lactams including cefotaxime, ceftazidime and aztreonam. Moreover, ESBLs and especially CTX-M variants were considered the major mechanisms involved in the resistance to cephalosporin antibiotics among Enterobacteriaceae, particularly, *E. coli* (Antonopoulos et al. 2019). Consequently, searching for β-lactamase inhibitors, particularly of natural sources, may be of great value.

Natural products such as essential oils and other plant constituents are promising source of alternative therapeutic agents (Abou El-Soud et al. 2015). The essential oil of *Ocimum basilicum* L. (Lamiaceae) showed antimicrobial activity by many researchers from different countries against different organisms (Hossain et al. 2010; Rattanachaikunsopon and Phumkhachorn 2010; Joshi 2014; Gaio et al. 2015). *O. basilicum* (basil) is one of the most popular and healthy culinary herbs. It is originally native to India and other Asian regions and is cultivated all over the world (Ahmed et al. 2019). Many investigations have been performed focussing on the chemical composition of the essential oil of *O. basilicum* (Costa et al. 2014; da Costa et al. 2015) and revealed a huge diversity in the oil constituents with different chemotypes from different regions of the world (Hassanpouraghdam et al. 2010). The variation in the chemical composition of basil essential oil largely affects the biological activities and consumer’s preference (Kiferle et al. 2019; Sousa et al. 2021). There is much research done on *O. basilicum* from different locations of Egypt (Ismail 2006; Abd El-Azim et al. 2015; Ahmed et al. 2019) except the central delta region. No reports were found in the literature regarding the β-lactamase inhibitory property of *O. basilicum*. Herein, we investigate, for the first time, the chemical composition, the spectrum of antimicrobial activity and β-lactamase inhibitory property of *O. basilicum* cultivated in Tanta (Center of Delta, Egypt). Additionally, *in silico* molecular docking of the most active component to the β-lactamase crystal structure, compared to clavulanic acid, was performed to explain the results.

## Materials and methods

### General

Camphor (PGVI) was purchased from VEB Laborchemie (Apolda, Germany).

Methanol used for extraction was of analytical grade (Fisher, Waltham, MA). Nuclear magnetic resonance (NMR) spectra were recorded using Bruker High Performance Digital FT-NMR Spectrometer Avance III 400 MHz (Bremen, Germany), operating at 400 MHz for ^1^H and 100 MHz for ^13^C NMR analyses.

### Plant material

The fresh aerial parts of *O. basilicum* were collected from the medicinal plant farm, Faculty of Pharmacy, Tanta University, in December 2018 and authenticated by Dr. Salma K. Shaltout, Botany Department, Faculty of Science, Tanta University. A voucher specimen has been kept in the herbarium of the Department of Pharmacognosy, Faculty of Pharmacy, Tanta University (PD-100-N). The plant material was dried in shade then reduced to a fine powder.

### Preparation of methanol extract

Dry powder of the aerial parts (100 g) was extracted by maceration in methanol at room temperature untill exhaustion. The methanol extract was filtered and evaporated using rotary evaporator under vacuum to yield 16.8% w/w. This extract was kept in a freezer for further biological and chemical analyses.

### Essential oil extraction

The essential oil was prepared from the dry powder of the aerial parts by hydrodistillation using a Clevenger type apparatus (Chenni et al. 2016). The yield of the prepared essential oil was 0.2% v/w of dry powder, which was stored in opaque sealed bottles under low temperature (4–5 °C) for GC/MS analysis and biological investigation.

### GC–MS of essential oil

The GC–MS is the most suitable technique for qualitative and quantitative characterization of the compounds of essential oils (Mesaros et al. 2009). A Thermo Scientific, Trace GC Ultra/ISQ Single Quadrupole MS (Waltham, MA) was used for the GC/MS analysis using a TG-5MS fused silica capillary column (30 mm, 0.25 mm, 0.25 mm film thickness). An electron ionization system with ionization energy of 70 eV was used for GC–MS detection. The carrier gas was Helium that used at a constant flow rate of 1 mL/min. The injector and MS transfer line temperature was set at 280 °C. The oven temperature was programmed at an initial temperature 40 °C (hold 3 min) to 280 °C as a final temperature at an increasing rate of 5 °C/min (hold 5 min). Identification of the compounds was performed based on the comparison of their retention index (RI) calculated with reference to a homologous series of *n*-alkanes (C8–C25) to that published in the literature, retention time (*R*_t_) and mass spectra with those of the NIST, WILLY library data of the GC/MS system. The RI was calculated using the Kovats retention index equation. The quantification of all the identified components was performed using a percent relative peak area.

### Isolation of methyl cinnamate

The aromatic water resulting from extraction of *O. basilicum* essential oil was concentrated and kept in the fridge for few days, white needle crystals were precipitated (yield 0.018% w/w). These crystals were filtered and stored in a cool place for spectral analysis and biological study.

### Isolation of cineol

To obtain cineol in a sufficient yield for antimicrobial testing, it was isolated from eucalyptus oil (TechnicoFlor, Allauch, France) by using the method which depends on the fact that cineole combines with acids as phosphoric acid to form loose compounds that are easily decomposed when treated with water (Mukherjee 2019). The isolated cineole (yield 80% w/w) was dehydrated over anhydrous sodium sulphate and subjected to NMR analysis to confirm its purity before using for antimicrobial study.

### UPLC-PDA-MS/MS analysis

UPLC analysis was run using a Nexera-I LC-2040 liquid chromatography system (Shimadzu, Kyoto, Japan). UPLC shimpack velox C18 column, 2.1 × 50 mm; 2.7 μm particle, was used with the following gradient (solvent A: water containing 0.1% formic acid; solvent B: acetonitrile) at flow rate of 0.2 mL/min: 0–2 min: 10% B; 2–5: linear gradient to 30% B; 5–15 min: linear gradient to 70% B; 15–22 min: linear gradient to 90% B; 22–25 min: linear gradient to 95% B; 25–26 min: linear gradient to 100% B; 26–29 min: isocratic 100% B; 29–30 min: linear gradient to 10% B. The sample (2 mg/mL) was prepared by dissolving the extract in HPLC methanol followed by filtration through 0.2 μm membrane disc filter, and the resultant solution was injected (3 μL) into the system. Detection was accomplished by using a LC-2030/2040 PDA detector and a LC-MS 8045 triple quadruple mass spectrometer equipped with an electrospray ionization (ESI) source in negative and positive mode (Shimadzu, Kyoto, Japan) applying the following settings: nebulizer gas N_2_, 3 L/min, 4 bar; dry gas N_2_, 10 L/min, 400 °C, capillary voltage (–4 kV); endplate offset (–4.5 kV); collision energy 8 eV (full MS) or 20–35 eV (MS/MS). Data processing was accomplished by using Shimadzu’s LabSolutions software (Kyoto, Japan). The compounds were identified based on the most intense adduct ion of each compound found in the full MS, MS/MS fragmentation ions, or neutral losses found in MS^2^ or full MS spectra and if possible, absorption *λ*_max_ from PDA spectra. Databases such as MassBank and FooDB were considered as references in the identification in addition to the published literature.

### Determination of the total content of flavonoids and polyphenols

Colorimetric assay was followed using the aluminium chloride method for determining the total flavonoid content (Kiranmai et al. 2011). Rutin (Sigma-Aldrich, St. Louis, MO) was used as a standard. The method of Folin-Ciocalteu and gallic acid (Sigma-Aldrich, St. Louis, MO) as a standard (Attard 2013) was adopted for measuring the total polyphenol content. The measured contents were expressed as mg/g equivalent of the corresponding standards.

### Strain and culture conditions

#### Reference strain

*E. coli* (ATCC 25922) was used as a quality control strain in the susceptibility test according to the European Committee on Antimicrobial Susceptibility Testing (EUCAST 2019).

#### Clinical isolates

A total of 10 multiple drug-resistant (MDR) *E. coli* clinical isolates were obtained from the Department of Pharmaceutical Microbiology, Faculty of Pharmacy, Tanta, University. Bacterial cultures were maintained in glycerol broth at −80 °C and were subcultured twice using Luria-Bertani agar before testing. All bacterial cultures were incubated aerobically for 24 h at 37 °C. They were previously identified using common biochemical tests including citrate utilization, oxidase test, growth on triple sugar iron, indole methyl red vogues proskauer citrate (IMViC) test and H_2_S production.

They were used to screen for ESBL. They were used for phenotypic detection of ESBL as well as genotypic detection of *bla*_CTX-M_ gene encoding for class A CTX-M.

#### Susceptibility test

Susceptibility of *E. coli* isolates to different antimicrobials was performed according to EUCAST (2019) to determine the resistance profile of each test isolate. The minimum inhibitory concentration (MIC) of each test product was determined according to the guidelines of EUCAST (2019) using broth microdilution assay in U-shaped 96-well microtiter plate via serial twofold dilutions to give a concentration range of 512–0.125 µg/mL. For oily product, it was emulsified in Mueller-Hinton broth using 2% DMSO, then mixed by vigorous shaking using vortex (Cazella et al. 2019). Medium-only was used as a negative control; however, wells containing bacteria-alone without drugs were used as a positive control of growth and also wells containing bacteria and 2% DMSO to confirm absence of the antimicrobial activity of the emulsifier at this concentration. Bacterial cells were inoculated through transferring 100 µL/well of 1 × 10^6^ CFU/mL that was diluted to give 1 × 10^5^ CFU/well. All microtiter plates were incubated for 18 ± 2 h, at 35 ± 1 °C. The value of MIC was defined as ‘the lowest concentration of test compound showed ≥90% reduction in OD_600_ compared to the control’.

### Screening for ESBL producing isolates

#### Double disc synergy test (DDST)

This test was used to screen for ESBL production among the MDR test isolates. Four antibiotic discs; cefepime, cefotaxime, aztreonam and ceftazidime were placed around a disc of amoxicillin/clavulanic acid at a distance of about 20 mm (centre to centre). Growth inhibition in the form of a keyhole is an indication of positive ESBL production (Apfalter et al. 2007; Tofteland et al. 2007).

#### Combination disc test

This is a confirmatory test that involves the use of cefotaxime and ceftazidime with and without clavulanic acid. The test discs used included: cefotaxime (30 μg), ceftazidime (30 μg), cefotaxime-clavulanic acid (30/10 μg) and ceftazidime-clavulanic acid (30/10 μg). These discs were transferred to MHA plates containing the test organism then were incubated at 37 °C for 24 h. After incubation, the diameter of the inhibition zone was measured and if it was found to be increased by ≥5 mm for either antibiotic combined with clavulanic acid, this was considered as evidence for the production of an ESBL (Schwaber et al. 2004; Jain and Mondal 2008).

#### Molecular detection of ESBL

Polymerase chain reaction was used for detection of antibiotic resistance genes among *E. coli* isolates which were positive ESBL in phenotypic tests. For DNA extraction, one colony of test bacteria was boiled in TE buffer for about 5 min followed by centrifugation, then the supernatant containing released DNA was considered as a PCR template. Taq DNA polymerase enzyme (Promega, Madison, WI) was utilized in the amplification reaction. The sequence of universal primers of *ctx–M* was as follows: MA1: SCSATGTGCAGYACCAGTAA, MA2: CCGCRATATGRTTGGTGGTG. Conditions of amplification reaction were: initial denaturation at 94 °C for 5 min; about 30 cycles of denaturation at 94 °C for 1 min, annealing temperature of 52 °C for 45 s, and elongation step at 72 °C for 1 min; then finally an elongation step at 72 °C for 10 min (Ramadan et al. 2019).

#### Preparation of cell free lysate from test isolates

Overnight culture of positive ESBL producing *E. coli* grown in MHB was subjected to centrifugation to harvest cells that were washed twice in phosphate buffer (PB, pH 7.4). Finally, cells were resuspended in PB and lysed using a probe ultrasonicator (Cole-Parmer, Vernon Hills, IL) 20 s four times with cooling intervals in between. Cell lysate was separated by centrifugation at 15,000×*g* for 30 min at 4 °C while the supernatant containing the ESBL was stored at −80 °C until use in β-lactamase activity assay (Dai et al. 2012).

#### β-Lactamase activity assay

β-Lactamase activity was measured colorimetrically using an β-lactamase inhibitor screening kit (catalogue number MAK222, Sigma-Aldrich, St. Louis, MO), which depends on the hydrolysis of nitrocefin (a chromogenic molecule and also a non-antimicrobial cephalosporin) by the test β-lactamase in the test material. This reaction leads to the formation of a coloured product. The later can be monitored through measuring the absorbance at 490 nm in a microtiter plate reader (Liu et al. 2018). This assay was done in presence and absence of different test botanical agents at 1/2 MIC and plates incubated in the dark for 10 min at 25 °C then subjected to enzymatic assay according to the manufacturer to screen for β-lactamase inhibitors. The best one was selected, and different concentrations were prepared to be tested for calculating the concentration that could inhibit 50% of the enzymatic activity (inhibitory concentration; IC_50_). The amount of β-lactamase enzyme needed to hydrolyse 1.0 μmol of substrate (nitrocefin) per minute at pH 7.0 at room temperature is equal to one unit of the enzyme. An online tool (IC_50_ Calculator-AAT Bioquist) was used to calculate the IC_50_ (https://www.aatbio.com/tools/ic50-calculator).

#### Docking study

The crystal structure of the β-lactamase in complex with piperacillin (E.C.3.5.2.6, PDB code: 3Q07) was downloaded from the protein databank. The complex is prepared with PrepWizard by adding missing hydrogen atoms, sidechains and loops. The hydrogen bond network was assigned for the amino acids at pH of 7.4. The protein–ligand complex was minimized to eliminate all clashes. The atomic coordinates of clavulanic acid were copied to the prepared structure from the crystal structure of TLA-1 S70G extended spectrum β-lactamase (PDB ID: 6PQ8). Then, the new complex was prepared for docking. The receptor grid was generated by Glide with default options using the location of clavulanic acid as the centroid of the docking box. Methyl cinnamate and clavulanic acid were prepared by LigPrep and docked with standard precision option into the clavulanic acid site. For each ligand, five poses were generated for analysis.

## Results

### GC–MS analysis

**Figure 1.**
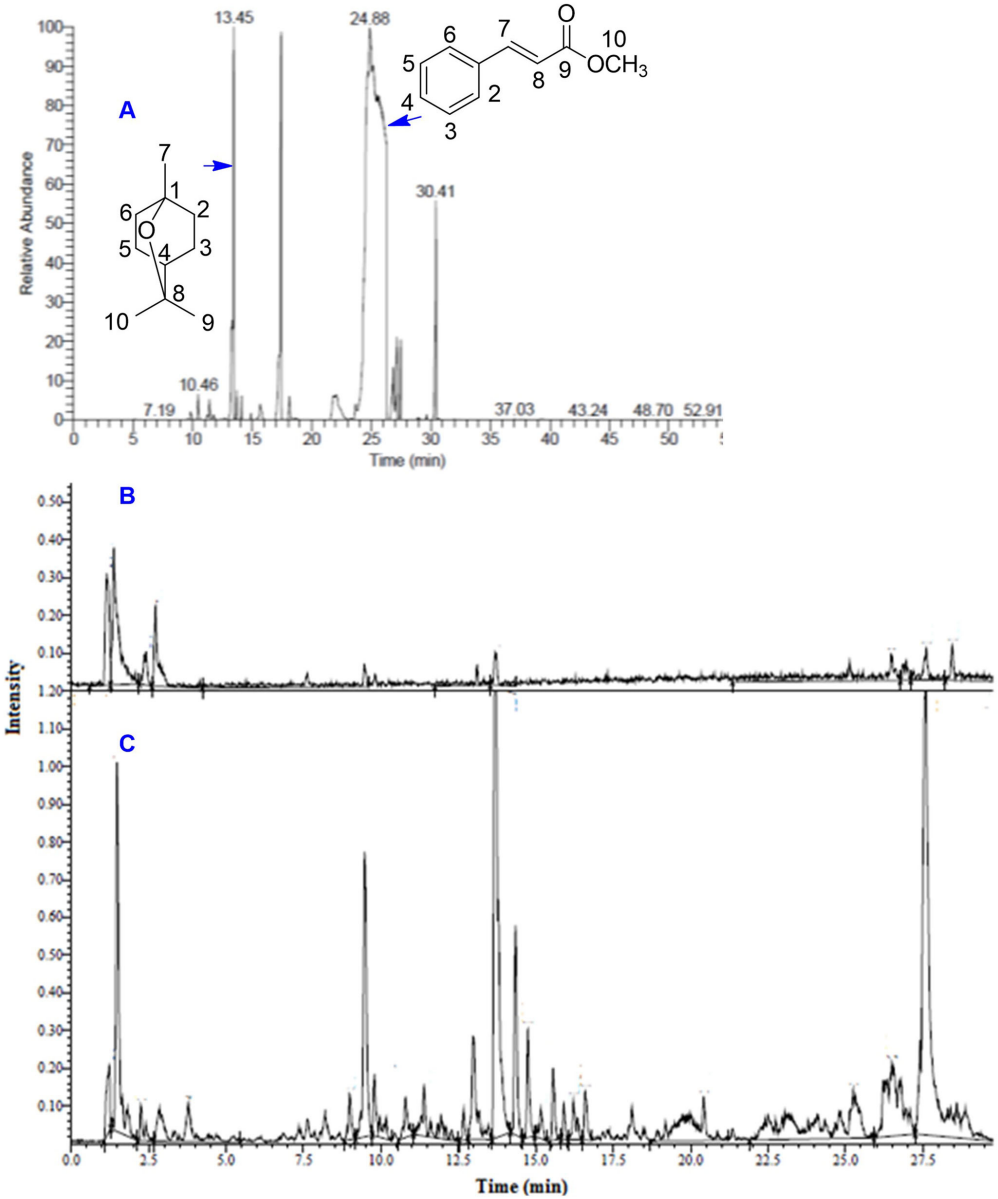
Analysis of essential oil and methanol extract of *O. basilicum*. (A) Gas chromatogram of *O. basilicum* essential oil with the structure of methyl cinnamate and 1,8-cineol linked to their respective peaks by arrows. UPLC-PDA-MS total ion chromatogram fingerprint of *O. basilicum* in (B) ESI + and (C) ESI– modes.

The GC/MS analysis of *O. basilicum* essential oil (Figure 1, panel A) revealed the identification of 20 compounds (Table 1) based on the comparison of their RI calculated with reference to a homologous series of *n*-alkanes (C8–C25) to that published in the literature, retention time (*R*_t_) and mass spectra with those of the NIST, WILLY library data of the GC/MS system. The RI was calculated using Kovats retention index equation and the data for an alkane mixture standard (C8–C25) were acquired and used for these determinations. The percentage of identified compounds was 95.78%. The terpene hydrocarbons represented 8.26%, 1.82% monoterpene and 6.44% sesquiterpenes. Oxygenated terpenoid compounds represented much higher % (43.66), 35.85% monoterpene and 7.81% sesquiterpenes. Non-terpenes (aromatic compounds) represented 43.86%. The major compound was found to be methyl cinnamate (Figure 1, panel A, 39.99%) followed by 1,8-cineol (17.73%) then camphor (14.61%).

**Table 1.** GC/MS of the essential oil of *Ocimum basilicum* L.

No.	RT	RI	Compound	Area %
1	9.84	944	α-Pinene	0.31
2	11.21	954	Camphene	0.12
3	11.40	979	Sabinene	0.61
4	11.75	996	α-Myrcene	0.18
5	13.43	1032	1,8-Cineol	17.73
6	13.70	1063	γ-Terpinene	0.70
7	14.90	1087	α-Terpinolene	0.14
8	15.68	1099	Linalool	0.78
9	17.45	1143	Camphor	14.61
10	18.14	1170	Borneol	0.77
11	18.56	1190	α-Terpineol	0.12
12	22.04	1210	Estragole	0.71
13	23.66	1345	α-Elemene	0.44
14	24.29	1359	Eugenol	1.60
15	24.55–26.26	1373	Methyl cinnamate	39.99
16	26.84	1380	α-Ylangene	2.72
17	27.15	1512	δ-Guaiene	3.28
18	27.84	1409	Methyl eugenol	2.27
19	29.66	1631	Cubenol	0.13
20	30.42	1649	α-Cadinol	7.68
Total percentage		95.78		
Identified components		Monoterpenes	Sesquiterpenes	Non-terpene
Oxygenated compounds		35.85	7.81	43.86
Non-oxygenated compounds		1.82	6.44	

RT: retention time; RI: retention index was compared to the published data.

Majors are highlighted in yellow.

### UPLC-PDA-MS/MS

Analysis of UPLC-PDA-MS/MS data in the negative and positive ion modes (Figure 1, panels B and C) identified 53 compounds (Table 2). Structures of identified compounds are shown in supplementary file.

**Table 2.** UPLC-PDA-MS/MS results for the methanol extract of *O. basilicum* L.

No.	*R*_t_ (min)	[M–H]^–^ *m/z*	[M + H]^+^*m/z*	MS^2^ ions *m/z*	Identification
1	1.49	335		173, 131, 101	3-*O*-Caffeoylshikimic acid (dactylifric acid) isomer
2	1.72	335		245, 181, 173, 141, 131, 101	3-*O*-Caffeoylshikimic acid (dactylifric acid)
3	4.11	197		197, 179, 135, 123, 109	3-(3,4-Dihydroxyphenyl) lactic acid Syringic acid; danshensu isomer
4	4.41	197		197, 179, 153, 135, 123, 119, 111, 96, 95	3-(3,4-Dihydroxyphenyl) lactic acid Syringic acid; danshensu
5	6.95	391		345, 258, 215, 185, 179, 161, 138, 119, 113	Unknown
6	7.01	391		345, 300, 220, 215, 193, 179, 149, 119, 113	Unknown
7	7.33	311		179, 149, 135, 87	Caftaric acid derivative
8	7.59		227	227, 209, 191, 173, 163, 155, 149, 137, 131, 121, 107, 105, 93, 85	Dihydroxy caffeic acid methyl ester
9	7.66	311		267, 179, 168, 149, 135, 103, 87, 85	Caftaric acid derivative
10	7.88	417		417, 152, 109	Salvianolic acid D/G
11	7.90	417		417, 241, 219, 152, 123, 108	Salvianolic acid D/G
12	7.90	387		387, 295, 219, 207, 196, 181, 163, 137, 119, 113	Hydroxy jasmonic acid-*O*-glucoside
13	7.99		411	411, 380, 361, 334, 305, 263, 249, 233, 214, 203, 187, 163,155, 147, 145, 135, 129, 109	Chlorogenic acid butyl ester
14	8.06		227		Dihydroxy caffeic acid methyl ester isomer
15	8.08	387		387, 207, 163, 119, 101, 89	Medioresinol
16	8.70	325		134, 117	Fertaric acid isomer
17	8.70	609		447, 285, 255, 193, 151, 125	Kaempferol-*O*-diglucoside
18	8.87	609		609, 301, 300, 285, 255, 193, 151, 125	Rutin
19	9.15	537		339, 313, 295, 269, 229, 203, 179, 159, 135, 109	Salvianolic acid I/H
20	9.35	537		295, 269, 229, 203, 197, 179, 159, 135, 109	Salvianolic acid I/H
21	9.49	325		148, 134, 119, 101, 87	Fertaric acid (feruloyl tartaric acid)
22	9.70		197	107, 179, 161, 143, 135, 133, 123, 119, 107, 105, 93	Trihydroxy cinnamic acid
23	9.84	717		475, 431, 365, 339, 321, 309, 267, 197, 161, 109	Rosmarinic acid dimer
24	9.96		163	163, 145, 135, 117, 107, 89	Methyl cinnamate
25	10.36		197	197, 179, 161, 151, 143, 137, 133, 123, 119, 107, 105, 93	Trihydroxy cinnamic acid isomer
26	10.43		163	163, 145, 135, 117, 107, 89	Methyl cinnamate isomer
27	10.43	359		197, 179, 161, 135, 133, 123	Rosmarinic acid isomer
28	10.46	717		269, 197, 179, 161, 150, 133	Salvianolic acid B/E
29	10.49	537		339, 313, 295, 269, 266, 251, 203, 197, 185, 179, 135, 109	*O-*Caffeoyl rosmarinic acid
30	10.69	359		197, 179, 161, 153, 135, 133, 123, 108	Rosmarinic acid
31	10.75	717		393, 357, 313, 283, 251, 225, 179, 163, 135	Salvianolic acid B/E
32	10.81	537		313, 295, 251, 197, 179, 161, 135	Lithospermic acid
33	11.26	493		313, 295, 277, 249, 203, 185, 159, 157, 135, 109	Salvianolic acid A
34	11.54	535		197, 177, 161, 135	Didehydrosalvianolic acid I/H
35	11.60	551		339, 321, 293, 277, 231, 229, 179, 135, 109	Methyl ester of salvianolic acid H/I ester
36	11.95	535		284, 253, 238, 221, 197, 177, 161, 135	Didehydrosalvianolic acid I/H
37	11.95	493		295, 197, 179, 159, 161, 135	Salvianolic acid A isomer
38	12.17	551		339, 239, 221, 193, 161, 135	Methyl ester of salvianolic acid H/I ester
39	12.48	327		327, 239, 229, 201, 185, 171, 156, 137, 127, 119, 97, 85	Methyl ester of salvianolic acid F
40	13.39	553		179, 135	Hydroxy salvianolic acid H/I
41	13.65	553		179, 135	Hydroxy salvianolic acid H/I
42	14.39	849		359, 311, 221, 197, 161, 135	Rosmarinic acid derivative
43	14.60	849		641, 507, 379, 359, 327, 309, 237, 197, 179, 161, 135	Rosmarinic acid derivative
44	14.77	313		313, 237, 229, 169, 147, 133, 123, 101, 99	Ladanein
45	15.07	313		297, 283, 269, 255, 229, 163	Cirsimaritin
46	15.33		181	181, 163, 145, 139, 135, 125, 121, 111, 109, 107, 95, 93	Caffeic acid
47	15.91		181	81, 163, 145, 140, 139, 135, 125, 124, 121, 111, 109, 107, 95, 93	Caffeic acid isomer
48	18.70		329	329, 314, 296, 268, 133	Salvigenin isomer
49	19.07		329	329, 314, 296, 268, 255, 237, 223, 208, 169, 135, 116, 105, 93	Salvigenin
50	20.08	329		314, 299, 271, 215, 199, 182, 126	Trihydroxy-octadecanoic acid
51	20.86	329		329, 293, 275, 237, 224, 209, 111, 97	Trihydroxy-octadecadienoic acid
52	26.76	455		455	Oleanolic acid
53	28.03	455		455	Oleanolic acid isomer

*R*_t_: retention time; *m/z*: mass to charge ratio; [M–H]^–^: deprotonated pseudomolecular ion; [M + H]^+^: protonated pseudomolecular ion.

Total flavonoid content was measured as 11.88 mg/g equivalent to rutin while the total polyphenols were calculated as 55.13 mg/g equivalent to gallic acid. This indicated that the extract is rich in polyphenols.

In the positive ion mode, 11 compounds were identified from which compounds **8**, **13**, **14**, **22** and **25** were reported for the first time in *O. basilicum*. Compounds **8** and **14** have an [M + H]^+^ ion at *m/z* 227 and were tentatively identified as dihydroxy caffeic acid methyl ester and its isomer. Compound **13** has an [M + H]^+^ ion at *m/z* 411 for chlorogenic acid butyl ester which was previously identified from *Osmanthus yunnanensis* (Franch.) P. S. Green (Oleaceae) (Wu et al. 2009). Compounds **22** and **25** showed an [M + H]^+^ ion at *m/z* 197 and were tentatively identified as trihydroxy cinnamic acid. Compounds **24** and **26** showed an [M + H]^+^ ion at *m/z* 163 for a methyl ester of cinnamic acid which was confirmed by GC analysis (Table 1) as one of the major volatile oils in our study. Compounds **46** and **47** exhibited an [M + H]^+^ ion at *m/z* 181 for caffeic acid and its isomer (Wu et al. 2009). Compounds **48** and **49** showed an [M + H]^+^ ion at *m/z* 329 for the flavonoid salvigenin and its isomer (Brijesh Kumar 2020).

In negative ion mode, 42 compounds were identified. Salvianolic acids and their derivatives were identified. Compounds **10** and **11** exhibited [M–H]^–^ ion at *m/z* 417 for salvianolic acid D/G (Wang et al. 2012; Farag et al. 2016). Compounds **20**, **29** and **32** gave an [M–H]^–^ ion at *m/z* 537 which indicated salvianolic acid H/I or isomers (Wang et al. 2012; Prinsi et al. 2019) and lithospermic acid (Taamalli et al. 2015; Farag et al. 2016). The didehydro-, hydroxy and methyl ester derivatives of salvianolic acid H/I with *m/z* 535, 553 and 551, respectively, were also tentatively identified (compounds **34**, **36**, **40**, **41**, **35** and **38**). Compounds **28** and **31** gave an [M–H]^–^ ion at *m/z* 717 characteristic for salvianolic acid E and salvianolic acid B (Wang et al. 2012; Farag et al. 2016; Prinsi et al. 2019). Compounds **33** and **37** gave an [M–H]^–^ ion at *m/z* 493 identical to salvianolic acid A (Prinsi et al. 2019). Compound **39** gave an [M–H]^–^ ion at *m/z* 327 and was tentatively identified as methyl ester of salvianolic acid F. Compounds **27** and **30** gave an [M–H]^–^ ion at *m/z* 359 for rosmarinic acid and its isomer (Hossain et al. 2010; Farag et al. 2016). Compounds **23**, **42** and **43** were identified as rosmarinic acid dimer and other derivatives. Flavonoid glycosides were represented by a diglucoside of kaempferol and rutin with an [M–H]^–^ ion at *m/z* 609, compounds **17** and **18** which were previously identified from *O. basilicum* (Farag et al. 2016; Prinsi et al. 2019; Brijesh Kumar 2020). Flavonoid aglycones were also identified. Compounds **44** and **45** gave an [M–H]^–^ ion at *m/z* 313 corresponding to cirsimaritin and ladenein (Koutsoulas et al. 2019).

Compounds **1** and **2** with *m/z* 335 were identified as dactylifric acid. Compounds **3** and **4** showed an [M–H]^–^ ion at *m/z* 197 for danshensu which is 3-(3,4-dihydroxyphenyl) lactic acid (Pink et al. 1994; Farag et al. 2016). Compounds **7** and **9** ([M–H]^–^ ion at *m/z* 311) typical for caftaric acid (Farag et al. 2016; Prinsi et al. 2019). Compound **12** exhibited an [M–H]^–^ ion at *m/z* 387 for medioresinol (Hossain et al. 2010). Compounds **16** and **21** showed an [M–H]^–^ ion at *m/z* 325 indicated feruloyl tartaric acid which is named fertaric acid and its isomer (Farag et al. 2016; Prinsi et al. 2019).

### NMR identification of methyl cinnamate isolated from *O. basilicum*

The ^1^H NMR spectrum (provided in supplementary file) indicated the presence of five aromatic protons suggesting a monosubstituted benzene ring. The ^13^C NMR spectrum (provided in supplementary file) showed eight signals accounting for 10 carbons. The aromatic carbons were represented by chemical shifts at *δ*_C_ 134.3 (C1), 128.8 (C2,6), 130.2 (C3,5) and 128.1 (C4), while the olefinic carbons (C7 and C8) exhibited chemical shifts at *δ*_C_ 117.2 and 144.9. Signals for carbonyl group at 167.8 (C9) and for deshielded alkyl carbon at *δ*_C_ 50.8 (C10) indicated an ester group. A methyl group peak at *δ*_H_ 3.79 confirmed a methyl ester. These spectral data are in good concordance with those in the literature for methyl cinnamate. The stereochemistry was identified as *E* based on the coupling constant for the trans protons *J*_H7–H8_=16 Hz (Spekreijse et al. 2012; Sitrallah et al. 2016).

### NMR identification of cineol isolated from eucalyptus oil

The ^1^H NMR spectrum (in CDCl_3_, provided in supplementary file) revealed a singlet at *δ*_H_ 1.06 integrating for a methyl group (position 7) and a singlet at *δ*_H_ 1.25 integrating for two methyl groups (positions 9 and 10). A broad singlet at *δ*_H_ 1.41 integrated for one proton at position 4. Three multiplet peaks at *δ*_H_ 1.50, 1.67 and 2.03 integrating for 4, 2 and 2 protons, respectively, were recorded. The multiplet at *δ*_H_ 1.50 was assigned to the endo protons at positions 2, 3, 5 and 6. The multiplet at *δ*_H_ 1.67 was assigned to the exo protons at positions 2 and 6, while the multiplet at *δ*_H_ 2.03 was ascribed to exo protons at positions 3 and 5. The ^13^C NMR spectrum (provided in supplementary file) exhibited *δ*_C_ at 23.5 (for C3 and C5), 27.9 (C7), 28.9 (C9 and C10), 31.6 (C2 and C6), 33.7 (C4), 69.9 (C1) and 73.7 (C8). The obtained data were matching those in literature for 1,8-cineol (Rinkel et al. 2016).

### Susceptibility test

As shown in Table 3, all test *E. coli* isolates were found to be MDR to 6–13 out of 17 tested antimicrobials. Furthermore, five different resistance patterns were detected among the test isolates. Additionally, susceptibility of *E. coli* isolates to different test natural products was carried out. Data revealed MIC values ranged between 8 and 64 µg/mL for both EO and methyl cinnamate. Furthermore, cineol and camphor showed MICs up to 128 µg/mL indicating little antimicrobial activity relative to EO (Table 4). For 2% DMSO, there was no antibacterial effect recorded.

**Table 3.** Resistance patterns of MDR *E. coli* test isolates.

Resistance pattern	No. of isolates displaying each pattern	No. of resistant markers	MAR index^a^
AMP-TZP-CXM-CIP-LEV-SXT	1	6	0.35
AMP-TZP-CXM-CIP-GAT-CN-AK	3	7	0.41
AMP-AMC-TZP-CXM-CTX-CIP-LEV-SXT	2	8	0.47
AMP-AMC-TZP-CXM-IMP-CIP-LEV-GAT-SXT-C	3	10	0.59
AMP-TZP-CL-CXM-CRO-CTX-FOX-CIP-CN-AK-SXT-TE-C	1	13	0.76

AMP: ampicillin; AMC: amoxicillin/clavulanate; TZP: piperacillin/tazobactam; CL: cefalexin; CXM: cefuroxime; CRO: ceftriaxone; CTX: cefotaxime; FOX: cefoxitin; CIP: ciprofloxacin; LEV: levofloxacin; GAT: gatifloxacin; CN: gentamicin; AK: amikacin; SXT: trimethoprim/sulfamethoxazole; IPM: imipenem; TE: tetracycline; C: chloramphenicol.

^a^ Regarding to the different 19 antimicrobial was tested and MAR index can be calculated by dividing the number of antimicrobials to which *E. coli* isolate was resistant by the total number of antimicrobials to which the isolate was exposed.

**Table 4.** Minimum inhibitory concentration of test products against *E. coli* isolates.

Test isolates	MIC (µg/mL)			
	EO	Cineol	Camphor	Methyl cinnamate
E1	16	128	128	32
E2	8	64	128	64
E3	8	128	128	64
E4	8	64	128	64
E5	8	128	128	64
E6	8	64	128	32
E7	16	128	128	64
E8	8	64	128	64
E9	8	128	128	64
E10	16	64	128	64
*E. coli* ATCC 25922	4	64	64	32

### β-Lactamase inhibitory activity

**Figure 2.**
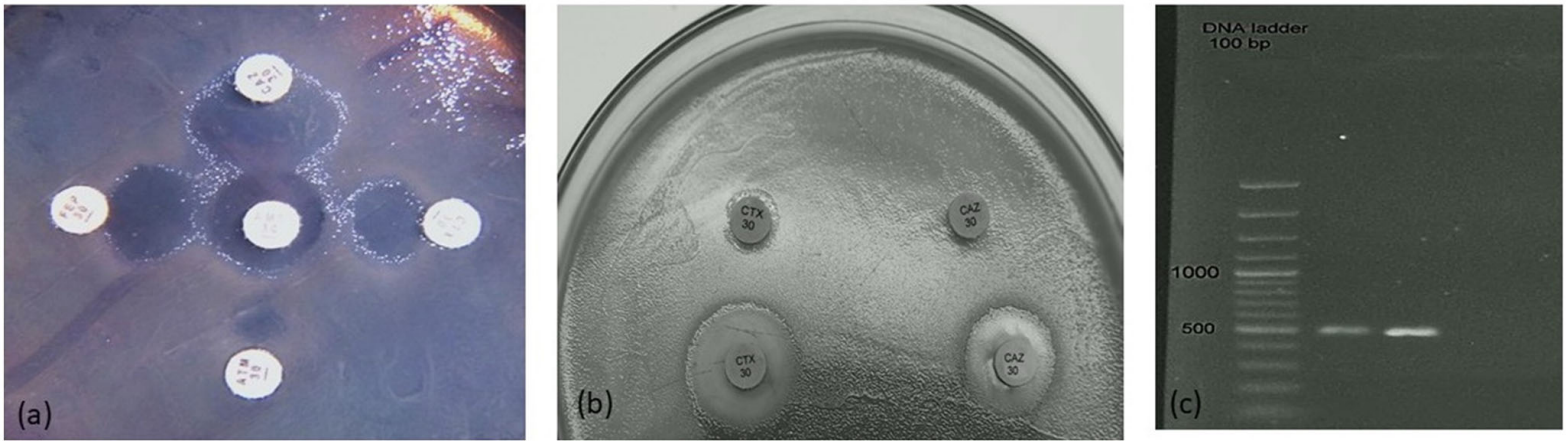
Phenotypic and genotypic screening for ESBL producing *E. coli* isolates using (a) double disc synergy test showing typical keyhole pattern denoting a positive ESBL producing isolate and (b) combination disc test showing increased zone diameter around the discs after addition of clavulanic acid confirming positive ESBL as referred by arrows. (c) Electrophoregram showing amplified amplicon bands at 500 bp denoting positive *bla*_CTX-M_ gene.

Phenotypic detection of ESBL activity among test *E. coli* pathogens showed only two positive isolates. Double disc synergy results usually observed as synergism between amoxicillin/clavulanic acid and the test antibiotic (Figure 2(a)). It showed typical keyhole pattern due to merged inhibition zones denoting a positive ESBL producing isolates. For confirmation, combination disc test was used. Data showed increased zone diameter (≥5 mm) around the discs of cefotaxime or ceftazidime after addition of clavulanic acid compared to their zone diameters when tested alone confirming positive ESBL as presented in Figure 2(b). Genotypic detection of *bla*_CTX-M_ gene revealed the presence of an amplified band at 500 bp in two test isolates as noticed in Figure 2(c).

**Figure 3.**
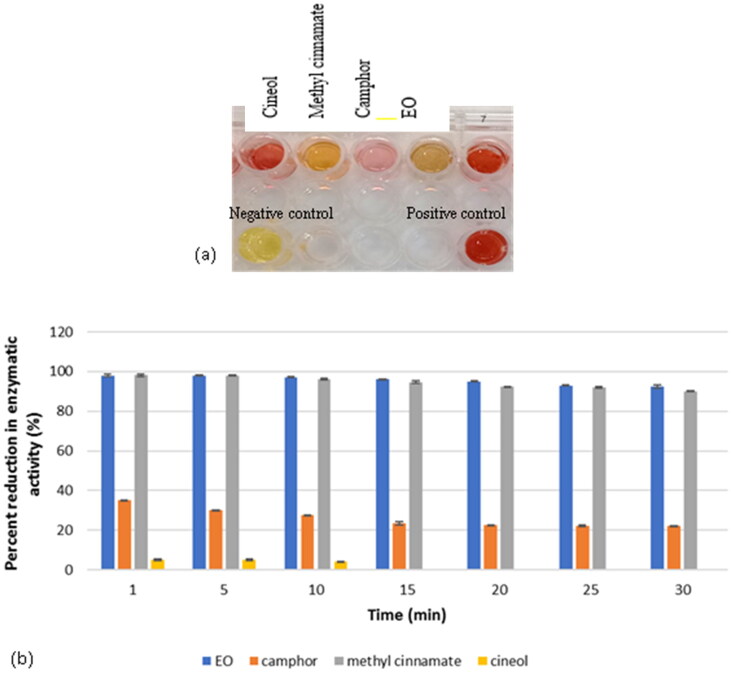
Screening for β-lactamase inhibitors among test botanical agents by colorimetric method showing (a) changes in colours due to different effects against test enzyme and (b) quantitative determination of percent reduction in enzymatic activity showing methyl cinnamate and EO were able to strongly inhibit β-lactamase activity.

Activity of β-lactamase enzyme was determined quantitatively using a colorimetric method. The test was done in the absence or presence of 1/2 MIC of the test agents to screen for enzymatic inhibitors (Figure 3(a)). Data showed that only methyl cinnamate and EO were the potent inhibitors where 55.1 and 59.07% inhibition was recorded, respectively, as shown in Figure 3(b).

**Figure 4.**
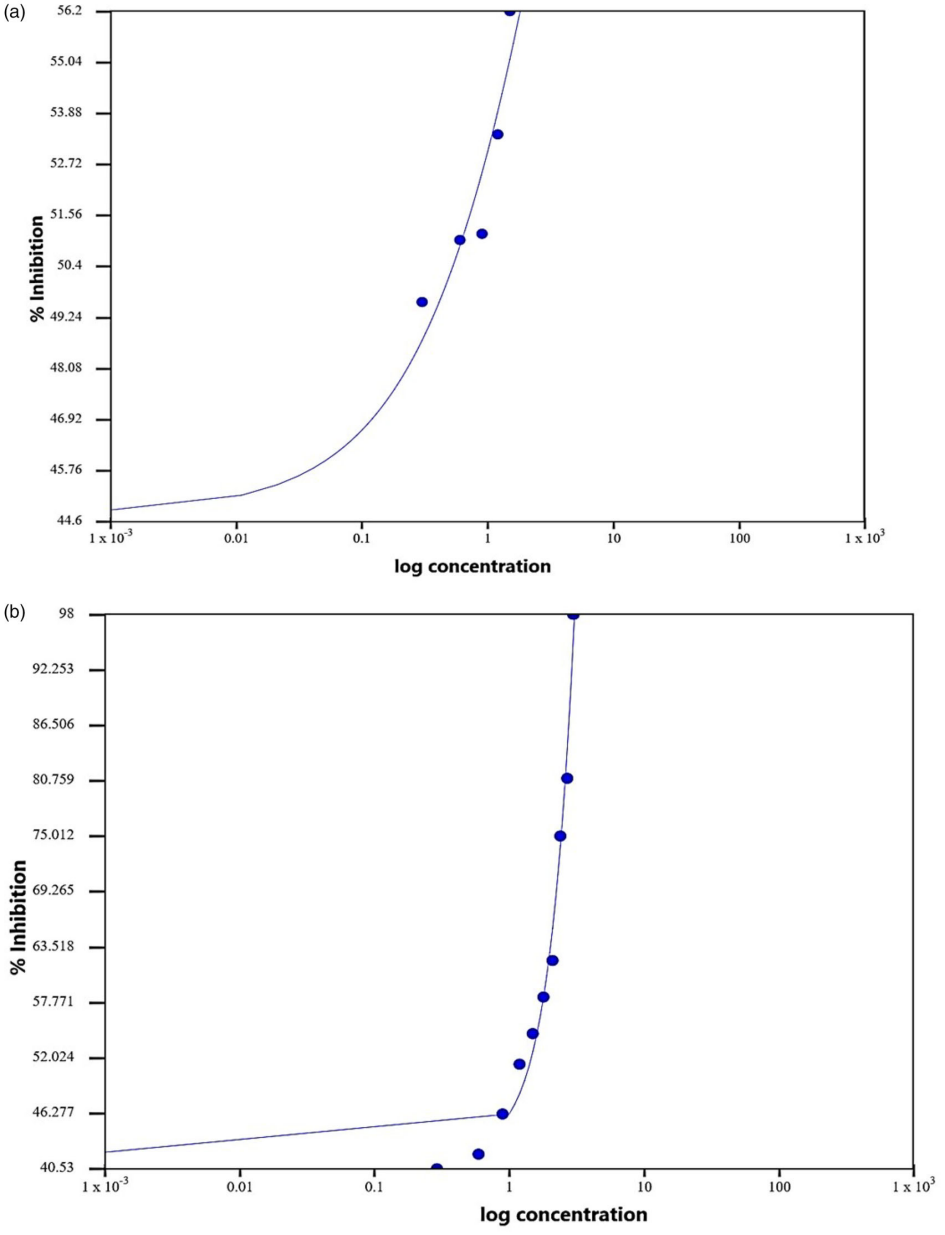
Representation of half maximal inhibitory concentration (IC_50_) values for both enzyme inhibitors: (a) methyl cinnamate as a test compound and (b) clavulanic acid as a standard β-lactamase inhibitor. Data presented as percent inhibition (%INH) of CTX-M against log the compound concentrations and nitrocefin as a substrate.

To calculate the IC_50_ of methyl cinnamate, different concentrations were evaluated against test enzyme (Figure 4 and Table 5). It was found that the concentration of methyl cinnamate required for 50% inhibition was 11.6 µg/mL vs. 8.1 µg/mL for clavulanic acid as a standard β-lactamase inhibitor (Figure 4 and Table 5).

**Table 5.** IC_50_ regression results for methyl cinnamate and clavulanic acid.

Parameter	Value methyl cinnamate	Value clavulanic acid
IC_50_	11.6008 (µg/mL)	8.1223 (µg/mL)
Equation	*Y* = 42.2462+$\frac{1467.5113\;-\;42.2462}{1\;+\;{(X/11.6008)\;}^{-2.4002}}$	*Y* = 44.789+$\frac{89.1376\;-\;44.789}{1\;+\;{(X/8.1223)\;}^{-0.7074}}$
Equation form	*Y* = Min+$\frac{\max\;-\;\min}{1\;+\;{(X/{\mathrm{IC}}_{50})\;}^{\text{Hill coefficient}}}$	*Y* = Min+$\frac{\max\;-\;\min}{1\;+\;{(X/{\mathrm{IC}}_{50})\;}^{\text{Hill coefficient}}}$

**Figure 5.**
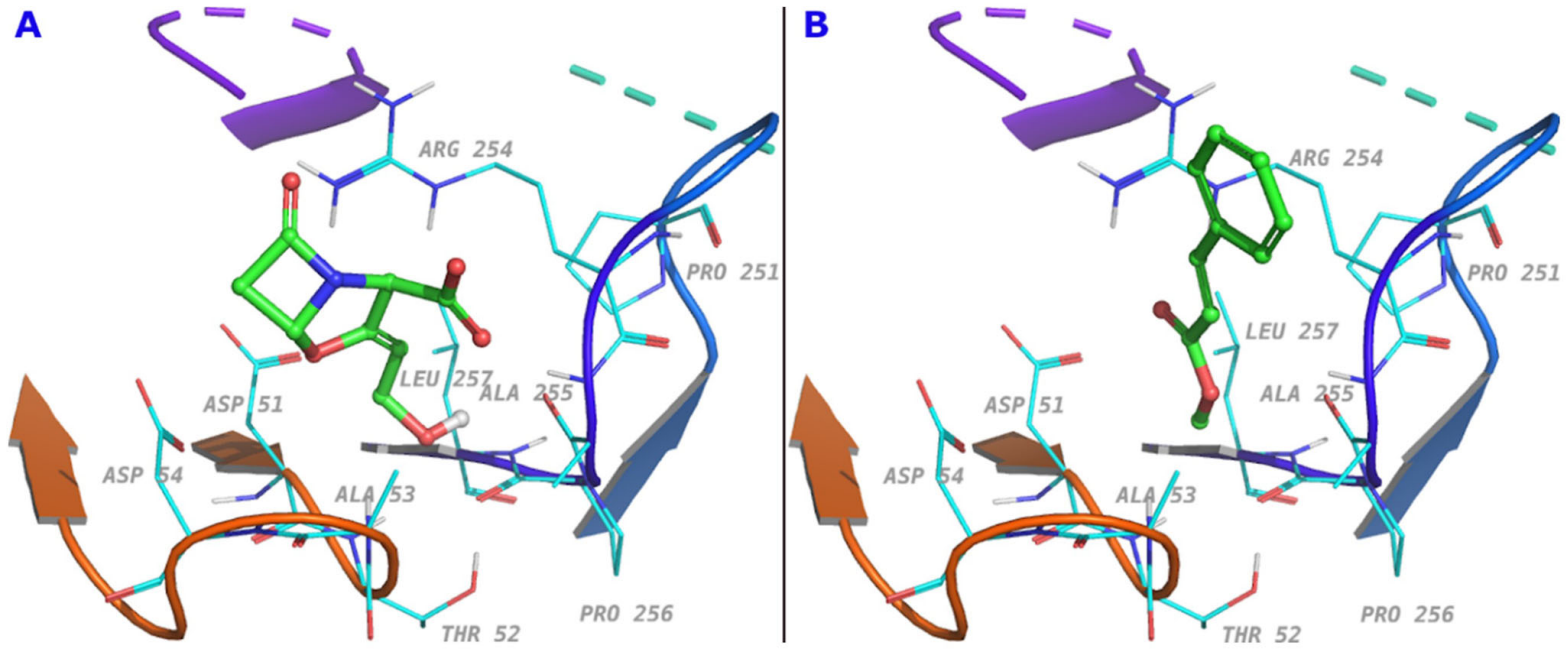
The amino acid residues involved in ligand interaction for (a) clavulanic acid and (b) methyl cinnamate.

*In silico* docking study was conducted for methyl cinnamate and clavulanic acid with the enzyme E.C.3.5.2.6, PDB code: 3Q07 which is the only enzyme of the class CTX-M with identified crystal structure and is the enzyme used in the Sigma-Aldrich kit (St. Louis, MO) used for our *in vitro* assay. Clavulanic acid docked with slightly better score (–4.8 kcal/mol vs. −3.2 kcal/mol for methyl cinnamate). The interaction with Arg254 was most profound through van der Waals, Coulombic and electrostatic types. The two compounds occupy the binding site similarly with key functional groups oriented to the same positions as shown in Figure 5.

## Discussion

Essential oils are one of the most important active constituents obtained from plants. They can be extracted by different methods, a famous one is distillation. Essential oils have historical applications as strong antimicrobial agents (Abers et al. 2021). The composition of the essential oil and the antimicrobial activity were previously studied for different plants of the *Ocimum* genus (Joshi 2013; Stefan et al. 2013; Salvi et al. 2022). According to the main components of the essential oils, four chemotypes have been distinguished: linalool-methyl chavicol, methyl chavicol, methyl cinnamate and eugenol. Another classification according to their aroma has been found such as sweet, lemon, cinnamon, camphor, anise and cloves (Pinto et al. 2019). The essential oil of *O. basilicum* cultivated in Egypt demonstrated high antimicrobial activity. The chemical composition of each oil showed some variations. Abd El-Azim et al. (2015) studied the oil of *O. basilicum* obtained from Aswan city (south Egypt). The abundant chemical constituents were linalool (33.9%) and eugenol (8.31%). The oil in that study demonstrated antimicrobial activity. Abou El-Soud et al. (2015) investigated *O. basilicum* which was purchased from local markets around Cairo city. The main components were linalool (48.4%), 1,8-cineol (12.2%), eugenol (6.6%) and methyl cinnamate (6.2%). That oil showed significant antifungal activity. Ahmed et al. (2019) evaluated the chemical composition of the essential oils of *O. basilicum* obtained from Assiut, Minia and Beni Suef of Egypt. The major constituents of the essential oils from the three locations were linalool, estragole and methyl cinnamate with some variation in the percentage. From these studies and others, it was accepted that the main constituent of basil is linalool. In our study, the major compound is methyl cinnamate. It is the first time to report that the main component of the essential oil of *O. basilicum* cultivated in Egypt is methyl cinnamate. This variation in the composition could be related to the ecological and environmental changes. The temperature in the Delta region is lower than in Cairo, Minia, Beni Suef and Aswan, also the soil composition is more fertile in this region. Thus, the stress factors are less which could have an impact on the constituents biosynthesized by the plant.

*O. basilicum* methanol extract was found rich in caffeic acid and rosmarinic acid derivatives. Salvianolic acids, esters of danshensu units and caffeic acid, are the major constituents and were found in different forms as free acids, esters or dehydro-derivatives. Dihydroxy caffeic acid methyl ester, chlorogenic acid butyl ester and trihydroxy cinnamic acid were detected for the first time in *O. basilicum*. Flavonoid content is mainly rutin, kaempferol-*O*-diglucoside, salvigenin, cirsimaritin and ladanein.

Methyl cinnamate with high quality (white needle crystals) was isolated here by a very simple procedure. Other researchers isolated it from plants by long chromatographic procedures (Ali et al. 2010; Park et al. 2020).

Development of antibiotic resistance is considered as an alarming public health concern, since it challenges the efficacy of antibiotic therapy. Therefore, screening for phytoactive molecules with antimicrobial activity is a preferable alternative due to safety and activity on multiple microbial targets (Walsh and Toleman 2012). In the present study, evaluation of the antimicrobial activities of *Ocimum* EO as well as other extracted and purified compounds was carried out against MDR *E. coli* isolates. Hendry et al. (2009) mentioned that 1,8-cineol displayed an antimicrobial activity against *E. coli* recording MIC value of 64 µg/mL. Also, Malapermal et al. (2017) reported antimicrobial activity of *Ocimum basilicum* oil. Additionally, Moo et al. (2021) attributed this effect to the ability of EO to increase the surface charge on the microbial cell beside increasing the permeability of the outer membrane resulting in leakage of proteins and nucleic acids. The present work showed that methyl cinnamate was the second in antimicrobial activity following EO denoting that the major activity of the later might be due to this compound. Stefanović et al. (2015) investigated the effect of methyl cinnamate against *E. coli* recording MIC value of >1000 µg/mL; however, our study showed lower MIC values indicating significant activity of ours and its possible application as antibacterial agent.

Recently, resistance of *E. coli* to antibiotics, particularly cephalosporins, has been increased due to extensive misuse of these drugs lowering their effectiveness leading to a health crisis (Li et al. 2018; Liu et al. 2018). Extended spectrum β-lactamases play an important role in the resistance mechanism of such pathogen. Accordingly, finding an effective drug that is able to inhibit the resistance of ESBL-producing *E. coli* became essential. In this work, *E. coli* clinical isolates were assessed for ESBL-production and it was found to produce CTX-M variant. All test agents were evaluated, at 1/2 MIC, for β-lactamase inhibitory effect by nitrocefin assay. Data showed that only EO and methyl cinnamate were the potent inhibitors. Hence, methyl cinnamate, as a pure compound, was selected to be evaluated at different concentrations to calculate its IC_50_. Results revealed that the IC_50_ for methyl cinnamate was 11.6 µg/mL vs. 8.1 µg/mL for clavulanic acid as a standard β-lactamase inhibitor. Furthermore, methyl cinnamate showed 98% reduction in the enzymatic activity at 1 mg/mL concentration (data not shown). Interestingly, it is the first report about the β-lactamase inhibitory activity of methyl cinnamate. Yang et al. (2020) stated that carnosic acid, a natural compound, did not possess direct antibacterial activity but demonstrated a significant inhibitory effect against NDM-1 recording an IC_50_ of 27.07 mM (Yang et al. 2020).

Docking of both clavulanic acid and methyl cinnamate to the β-lactamase enzyme E.C.3.5.2.6, a member of CTX-M-9 family, indicated similar type of interaction in the enzyme binding site with a slight difference in the binding energy. Both methyl cinnamate and clavulanic acid exhibited binding to Arg254 which imparts a positive charge at the active site. According to Bethel et al., a positive charge from an arginine residue at the active site of CTX-M-9 lactamases is essential for the recognition of the carboxylate group of the inhibitors/substrates (Bethel et al. 2011) and thus plays an important role in its mechanism of action. In our model, the residue Arg254 could play the same role and hence explains the inhibitory effect of methyl cinnamate. This result supports our *in vitro* findings.

## Conclusions

*O. basilicum*, from the central Delta of Egypt, exhibited *in vitro* β-lactamase inhibitory activity. Methyl cinnamate, the major component of essential oil in our plant, showed an IC_50_ close to that of clavulanic acid which was supported by *in silico* molecular docking. This activity was attributed to methyl cinnamate, the major essential oil in our plant; hence, future studies are required for more insights into the mechanism and kinetics involved.

## Supplementary Material

Supplemental Material

## References

[CIT0001] Abd El-Azim MH, Abdelgawad AA, El-Gerby M, Ali S, El-Mesallamy A. 2015. Chemical composition and antimicrobial activity of essential oil of Egyptian *Ocimum basilicum* L. Indo Am J Pharm Sci. 2:837–842.

[CIT0002] Abers M, Schroeder S, Goelz L, Sulser A, Rose TS, Puchalski K, Langland J. 2021. Antimicrobial activity of the volatile substances from essential oils. BMC Complement Med Ther. 21(1):1–14. 33865375 10.1186/s12906-021-03285-3 PMC8053297

[CIT0003] Abou El-Soud NH, Deabes M, Abou El-Kassem L, Khalil M. 2015. Chemical composition and antifungal activity of *Ocimum basilicum* L. essential oil. Open Access Maced J Med Sci. 3(3):374–379. 27275253 10.3889/oamjms.2015.082 PMC4877822

[CIT0005] Ahmed AF, Attia FA, Liu Z, Li C, Wei J, Kang W. 2019. Antioxidant activity and total phenolic content of essential oils and extracts of sweet basil (*Ocimum basilicum* L.) plants. Food Sci Hum Wellness. 8(3):299–305.

[CIT0006] Ali N, Rahmani M, Shaari K, Ali A, Lian GEC. 2010. Antimicrobial activity of *Cinnamomum impressicostatum* and *C*. *pubescens* and bioassay-guided isolation of bioactive (E)-methyl cinnamate. J Biol Sci. 10(2):101–106.

[CIT0007] Antonopoulos DA, Assaf R, Aziz RK, Brettin T, Bun C, Conrad N, Davis JJ, Dietrich EM, Disz T, Gerdes S, et al. 2019. PATRIC as a unique resource for studying antimicrobial resistance. Brief Bioinform. 20(4):1094–1102. 28968762 10.1093/bib/bbx083 PMC6781570

[CIT0008] Apfalter P, Assadian O, Daxböck F, Hirschl AM, Rotter ML, Makristathis A. 2007. Extended double disc synergy testing reveals a low prevalence of extended-spectrum β-lactamases in *Enterobacter* spp. in Vienna, Austria. J Antimicrob Chemother. 59(5):854–859. 17347178 10.1093/jac/dkm060

[CIT0010] Attard E. 2013. A rapid microtitre plate Folin-Ciocalteu method for the assessment of polyphenols. Open Life Sci. 8(1):48–53.

[CIT0012] Bethel CR, Taracila M, Shyr T, Thomson JM, Distler AM, Hujer KM, Hujer AM, Endimiani A, Papp-Wallace K, Bonnet R, et al. 2011. Exploring the inhibition of CTX-M-9 by beta-lactamase inhibitors and carbapenems. Antimicrob Agents Chemother. 55(7):3465–3475. 21555770 10.1128/AAC.00089-11 PMC3122419

[CIT0013] Brijesh Kumar VB, Tiwari S, Pandey R. 2020. Phytochemistry of plants of genus *Ocimum*. Florida (FL): CRC Press, Taylor & Francis Group.

[CIT0014] Cazella LN, Glamoclija J, Sokovic M, Gonçalves JE, Linde GA, ’Colauto NB, Gazim ZC. 2019. Antimicrobial activity of essential oil of *Baccharis dracunculifolia* DC (Asteraceae) aerial parts at flowering period. Front Plant Sci. 10:27. 30761171 10.3389/fpls.2019.00027 PMC6361755

[CIT0015] Chenni M, El Abed D, Rakotomanomana N, Fernandez X, Chemat F. 2016. Comparative study of essential oils extracted from Egyptian basil leaves (*Ocimum basilicum* L.) using hydro-distillation and solvent-free microwave extraction. Molecules. 21(1):113–128. 10.3390/molecules21010113 PMC6273689 26797599

[CIT0016] Costa A, Arrigoni-Blank MDF, Silva M, Alves MF, Santos DDA, Alves PB, Blank AF. 2014. The impact of hybridization on the volatile and sensorial profile of *Ocimum basilicum* L. ScientificWorldJournal. 2014:824594. 24558334 10.1155/2014/824594 PMC3914388

[CIT0017] da Costa AS, Arrigoni-Blank M, Carvalho F, de Santana ADD, Santos DDA, Alves PB, Blank AF. 2015. Chemical diversity in basil (*Ocimum* sp.) germplasm. ScientificWorldJournal. 2015:352638. 25629084 10.1155/2015/352638 PMC4299303

[CIT0018] Dai X, Xiang S, Li J, Gao Q, Yang K. 2012. Development of a colorimetric assay for rapid quantitative measurement of clavulanic acid in microbial samples. Sci China Life Sci. 55(2):158–163. 22415687 10.1007/s11427-012-4287-x

[CIT0019] EUCAST. 2019. Breakpoint tables for interpretation of MICs and zone diameters. Version 9.0. http://www.eucast.org/ast_of_bacteria/previous_versions_of_documents/.

[CIT0021] Fam N, Leflon-Guibout V, Fouad S, Aboul-Fadl L, Marcon E, Desouky D, El-Defrawy I, Abou-Aitta A, Klena J, Nicolas-Chanoine MH. 2011. CTX-M-15-producing *Escherichia coli* clinical isolates in Cairo (Egypt), including isolates of clonal complex ST10 and clones ST131, ST73, and ST405 in both community and hospital settings. Microb Drug Resist. 17(1):67–73. 21128836 10.1089/mdr.2010.0063

[CIT0022] Farag M, Ezzat S, Salama MM, Tadros M. 2016. Anti-acetylcholinesterase potential and metabolome classification of 4 *Ocimum* species as determined via UPLC/qTOF/MS and chemometric tools. J Pharm Biomed Anal. 125:292–302. 27061877 10.1016/j.jpba.2016.03.037

[CIT0024] Gaio I, Saggiorato AG, Treichel H, Cichoski AJ, Astolfi V, Cardoso RI, Toniazzo G, Valduga E, Paroul N, Cansian RL. 2015. Antibacterial activity of basil essential oil (*Ocimum basilicum* L.) in Italian-type sausage. J Verbr Lebensm. 10(4):323–329.

[CIT0026] Hassanpouraghdam MB, Gohari GR, Tabatabaei SJ, Dadpour MR. 2010. Inflorescence and leaves essential oil composition of hydroponically grown *Ocimum basilicum* L. J Serb Chem Soc. 75(10):1361–1368.

[CIT0027] Hendry ER, Worthington T, Conway BR, Lambert PA. 2009. Antimicrobial efficacy of eucalyptus oil and 1,8-cineole alone and in combination with chlorhexidine digluconate against microorganisms grown in planktonic and biofilm cultures. J Antimicrob Chemother. 64(6):1219–1225. 19837714 10.1093/jac/dkp362

[CIT0028] Hossain MA, Kabir M, Salehuddin S, Rahman SM, Das A, Singha SK, Alam MK, Rahman A. 2010. Antibacterial properties of essential oils and methanol extracts of sweet basil *Ocimum basilicum* occurring in Bangladesh. Pharm Biol. 48(5):504–511. 20645791 10.3109/13880200903190977

[CIT0030] Ismail M. 2006. Central properties and chemical composition of *Ocimum basilicum* essential oil. Pharm Biol. 44(8):619–626.

[CIT0031] Jain A, Mondal R. 2008. TEM & SHV genes in extended spectrum β-lactamase producing *Klebsiella* species & their antimicrobial resistance pattern. Indian J Med Res. 128(6):759–764. 19246801

[CIT0032] Joshi RK. 2013. Chemical composition, *in vitro* antimicrobial and antioxidant activities of the essential oils of *Ocimum gratissimum*, *O. sanctum* and their major constituents. Indian J Pharm Sci. 75(4):457–462. 24302801 10.4103/0250-474X.119834 PMC3831728

[CIT0033] Joshi RK. 2014. Chemical composition and antimicrobial activity of the essential oil of *Ocimum basilicum* L. (sweet basil) from Western Ghats of North West Karnataka, India. Anc Sci Life. 33(3):151–156. 25538349 10.4103/0257-7941.144618 PMC4264302

[CIT0034] Kiferle C, Ascrizzi R, Martinelli M, Gonzali S, Mariotti L, Pistelli L, Flamini G, Perata P. 2019. Effect of iodine treatments on *Ocimum basilicum* L.: biofortification, phenolics production and essential oil composition. PLOS One. 14(12):e0226559. 31841559 10.1371/journal.pone.0226559 PMC6913995

[CIT0035] Kiranmai M, Kumar CM, Mohammed I. 2011. Comparison of total flavanoid content of *Azadirachta indica* root bark extracts prepared by different methods of extraction. Res J Pharm Biol Chem Sci. 2:254–261.

[CIT0036] Koutsoulas A, Čarnecká M, Slanina J, Tóth J, Slaninová I. 2019. Characterization of phenolic compounds and antiproliferative effects of *Salvia pomifera* and *Salvia fruticosa* extracts. Molecules. 24(16):2921–2938. 10.3390/molecules24162921 PMC6720736 31408993

[CIT0037] Li Y, Sun Q-l, Shen Y, Zhang Y, Yang J-w, Shu L-b, Zhou H-w, Wang Y, Wang B, Zhang R, et al. 2018. Rapid increase in prevalence of carbapenem-resistant Enterobacteriaceae (CRE) and emergence of colistin resistance gene mcr-1 in CRE in a hospital in Henan. J Clin Microbiol. 56(4):e01932. 29386265 10.1128/JCM.01932-17 PMC5869811

[CIT0039] Liu S, Zhou Y, Niu X, Wang T, Li J, Liu Z, Wang J, Tang S, Wang Y, Deng X. 2018. Magnolol restores the activity of meropenem against NDM-1-producing *Escherichia coli* by inhibiting the activity of metallo-beta-lactamase. Cell Death Discov. 4:1–8. 10.1038/s41420-018-0029-6 PMC5841300 29531825

[CIT0040] Mesaros C, Culea M, Iordache A, Cozar O. 2009. GC–MS characterization of the compounds in some essential oils. Bull UASVM Agric. 66:111–116.

[CIT0041] Malapermal V, Botha I, Krishna S, Mbatha J. 2017. Enhancing antidiabetic and antimicrobial performance of *Ocimum basilicum*, and *Ocimum sanctum* (L.) using silver nanoparticles. Saudi J Biol Sci. 24(6):1294–1305. 28855825 10.1016/j.sjbs.2015.06.026 PMC5562380

[CIT0042] Moo CL, Osman MA, Yang SK, Yap WS, Ismail S, Lim SHE, Chong CM, Lai KS. 2021. Antimicrobial activity and mode of action of 1,8-cineol against carbapenemase-producing *Klebsiella pneumoniae*. Sci Rep. 11:20824. 34675255 10.1038/s41598-021-00249-y PMC8531306

[CIT0043] Mukherjee PK. 2019. Quality control and evaluation of herbal drugs: evaluating natural products and traditional medicine. Oxford (UK): Elsevier.

[CIT0044] Pinto J, Blank A, Nogueira PC, Arrigoni-Blank MdF, Andrade T, Sampaio TS, Pereira K. 2019. Chemical characterization of the essential oil from leaves of basil genotypes cultivated in different seasons. Bol Latinoam Caribe Plant Med Aromat. 18(1):58–70.

[CIT0045] Park KR, Lee H, Cho M, Yun HM. 2020. A phytochemical constituent, (*E*)-methyl-cinnamate isolated from *Alpinia katsumadai* Hayata suppresses cell survival, migration, and differentiation in pre-osteoblasts. Int J Mol Sci. 21(10):3700–3713. 10.3390/ijms21103700 PMC7279157 32456334

[CIT0046] Pink D, Naczk M, Baskin K, Shahidi F. 1994. Theoretical analysis of ultraviolet–visible spectra of various phenolic acid fractions of canola. J Agric Food Chem. 42(6):1317–1322.

[CIT0047] Prinsi B, Morgutti S, Negrini N, Faoro F, Espen L. 2019. Insight into composition of bioactive phenolic compounds in leaves and flowers of green and purple basil. Plants. 9(1):22–37. 10.3390/plants9010022 PMC7020237 31877999

[CIT0048] Ramadan AA, Abdelaziz NA, Amin MA, Aziz RK. 2019. Novel blaCTX-M variants and genotype-phenotype correlations among clinical isolates of extended spectrum beta lactamase producing *Escherichia coli.* Sci Rep. 9(1):4224. 30862858 10.1038/s41598-019-39730-0 PMC6414621

[CIT0049] Rattanachaikunsopon P, Phumkhachorn P. 2010. Antimicrobial activity of basil (*Ocimum basilicum*) oil against *Salmonella enteritidis in vitro* and in food. Biosci Biotechnol Biochem. 74(6):1200–1204. 20530897 10.1271/bbb.90939

[CIT0050] Rinkel J, Rabe P, Zur HL, Dickschat JS. 2016. A detailed view on 1,8-cineol biosynthesis by *Streptomyces clavuligerus*. Beilstein J Org Chem. 12:2317–2324. 28144299 10.3762/bjoc.12.225 PMC5238540

[CIT0051] Salvi P, Gulshan K, Nishu G, Ashish V, Sivasubramanian R, Nilesh R, Vibhav G. 2022. Antimicrobial potential of essential oils from aromatic plant *Ocimum* sp.: a comparative biochemical profiling and *in-silico* analysis. Agronomy. 12(3):627–644.

[CIT0052] Schwaber MJ, De-Medina T, Carmeli Y. 2004. Epidemiological interpretation of antibiotic resistance studies–what are we missing? Nat Rev Microbiol. 2(12):979–983. 15550943 10.1038/nrmicro1047

[CIT0054] Sitrallah S, Merza J, Homs S. 2016. Isolation and identification of methyl cinnamate from Syrian *Ocimum basilicum*. Chem Mater Res. 8(6):13–19.

[CIT0055] Sousa A, Silva C, Costa-Júnior H, Silva N, Pinto J, Blank A, Soares A, Costa-Júnior L. 2021. Essential oils from *Ocimum basilicum* cultivars: analysis of their composition and determination of the effect of the major compounds on *Haemonchus contortus* eggs. J Helminthol. 95:E17. 33745470 10.1017/S0022149X21000080

[CIT0056] Spekreijse J, Le Nôtre J, van Haveren J, Scott EL, Sanders JP. 2012. Simultaneous production of biobased styrene and acrylates using ethenolysis. Green Chem. 14(10):2747–2751.

[CIT0057] Stefan M, Zamfirache MM, Padurariu C, Trută E, Gostin I. 2013. The composition and antibacterial activity of essential oils in three *Ocimum* species growing in Romania. Central Eur J Biol. 8(6):600–608.

[CIT0058] Stefanović OD, Radojević ID, Čomić LR. 2015. Synthetic cinnamates as potential antimicrobial agents. Hem Ind. 69:37–42.

[CIT0059] Taamalli A, Arráez-Román D, Abaza L, Iswaldi I, Fernández-Gutiérrez A, Zarrouk M, Segura-Carretero A. 2015. LC–MS-based metabolite profiling of methanolic extracts from the medicinal and aromatic species *Mentha pulegium* and *Origanum majorana*. Phytochem Anal. 26(5):320–330. 25982347 10.1002/pca.2566

[CIT0060] Tofteland S, Haldorsen B, Dahl KH, Simonsen GS, Steinbakk M, Walsh TR, Sundsfjord A, Norwegian ESBL Study Group. 2007. Effects of phenotype and genotype on methods for detection of extended-spectrum-β-lactamase-producing clinical isolates of *Escherichia coli* and *Klebsiella pneumoniae* in Norway. J Clin Microbiol. 45(1):199–205. 17079502 10.1128/JCM.01319-06 PMC1828980

[CIT0061] Walsh TR, Toleman MA. 2012. The emergence of pan-resistant Gram-negative pathogens merits a rapid global political response. J Antimicrob Chemother. 67(1):1–3. 21994911 10.1093/jac/dkr378

[CIT0062] Wang S, Liu L, Wang L, Hu Y, Zhang W, Liu R. 2012. Structural characterization and identification of major constituents in Jitai tablets by high-performance liquid chromatography/diode-array detection coupled with electrospray ionization tandem mass spectrometry. Molecules. 17(9):10470–10493. 22945027 10.3390/molecules170910470 PMC6268525

[CIT0063] Wu ZJ, Ma XL, Fang DM, Qi HY, Ren WJ, Zhang GL. 2009. Analysis of caffeic acid derivatives from *Osmanthus yunnanensis* using electrospray ionization quadrupole time-of-flight mass spectrometry. Eur J Mass Spectrom. 15(3):415–429. 10.1255/ejms.992 19395777

[CIT0064] Yang Y, Guo Y, Zhou Y, Gao Y, Wang X, Wang J, Niu X. 2020. Discovery of a novel natural allosteric inhibitor that targets NDM-1 against *Escherichia coli*. Front Pharmacol. 11:1531. 10.3389/fphar.2020.581001 PMC7566295 33123013

