# Supplementary material for: In vitro and in silico β-lactamase inhibitory properties and phytochemical profile of Ocimum basilicum cultivated in central delta of Egypt
Source: Pharm Biol. 2022 Oct 13;60(1):1969–80. doi: 10.1080/13880209.2022.2127791 (PMC9578474; doi:10.1080/13880209.2022.2127791)
Supplement: Supplemental Material [file IPHB_A_2127791_SM6167.docx]

***In* *Vitro* and *In* *Silico* β- Lactamase Inhibitory Properties and Phytochemical Profile of *Ocimum basilicum* L. Cultivated in Central Delta of Egypt**

Nagwa A. Shoeib^a^, Lamiaa A. Al-Madboly^b^, Amany E. Ragab^a,^*

^a^ Department of Pharmacognosy, Tanta University, Tanta (31527), Egypt

^b^ Department of Pharmaceutical Microbiology, Tanta University, Tanta (31527), Egypt

*Correspondence: Amany E Ragab (amany.ragab@pharm.tanta.edu.eg)

Figure S1: Structures identified by UPLC-PDA-MS/MS analysis.


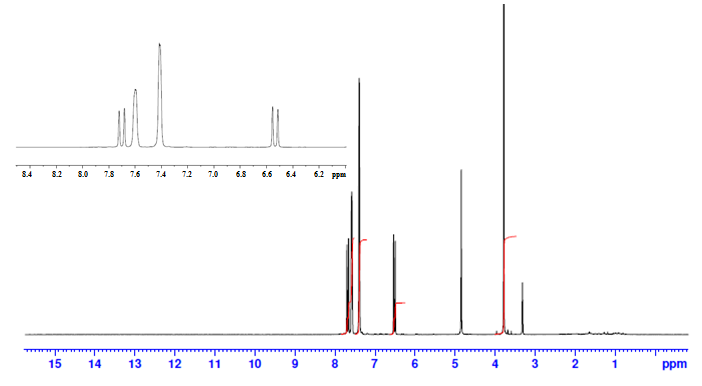


Figure S2: ^1^HNMR spectrum of methyl cinnamate (CD_3_OD, 400 MHz).


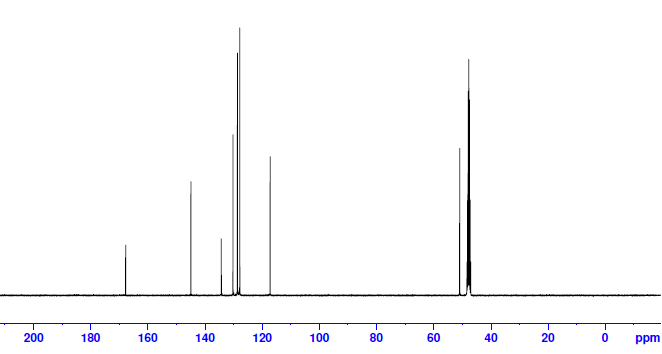


Figure S3: ^13^CNMR spectrum of methyl cinnamate (CD_3_OD, 100 MHz).

Figure S4: ^1^HNMR spectrum of 1,8-cineol (CDCl_3_, 400 MHz).

Figure S5: ^13^CNMR spectrum of 1,8-cineol (CDCl_3_, 100 MHz).
